# Catalysis of electro-oxidation of antibiotics by nitroxyl radicals and the electrochemical sensing of vancomycin†

**DOI:** 10.1039/d1ra03681e

**Published:** 2021-06-18

**Authors:** Tetsuya Ono, Kyoko Sugiyama, Sachiko Komatsu, Masayuki Kumano, Kentaro Yoshida, Takenori Dairaku, Tsutomu Fujimura, Yusuke Sasano, Yoshiharu Iwabuchi, Yoshitomo Kashiwagi, Katsuhiko Sato

**Affiliations:** School of Pharmaceutical Sciences, Ohu University 31-1 Misumido, Tomita-machi Koriyama Fukushima 963-8611 Japan t-ono@pha.ohu-u.ac.jp; Faculty of Pharmaceutical Science, Tohoku Medical and Pharmaceutical University 4-4-1 Komatsushima, Aoba Sendai Miyagi 981-8558 Japan satok@tohoku-mpu.ac.jp; Graduate School of Pharmaceutical Sciences, Tohoku University 6-3 Aoba, Aramaki Aoba-ku Sendai 980-8578 Japan; Department of Creative Engineering, National Institute of Technology, Tsuruoka College 104 Sawada, Inooka Tsuruoka Yamagata 997-8511 Japan

## Abstract

Quantifying drug concentrations *in vivo* quickly and easily is possible using electrochemical methods. The present study describes the electrochemical detection of vancomycin (VCM) and other antibiotics from the current obtained using nitroxyl radicals as electrocatalysts. Nortropine *N*-oxyl (NNO), which is more active than 2,2,6,6-tetramethylpiperidine 1-oxyl (TEMPO), a typical nitroxyl radical compound, produced greater current values for drugs with intramolecular hydroxy groups and secondary and tertiary amines. However, because the catalytic action of NNO is inactivated by primary amines in the substrate, VCM and teicoplanin with primary amines could not be detected. TEMPO was less active than NNO but not inactivated against primary amines. Therefore, electrochemical sensing of vancomycin was done using 4-acetamido-2,2,6,6-tetramethylpiperidine 1-oxyl (A-TEMPO), which has a greater oxidation capacity than TEMPO due to its electron-withdrawing groups. As a result, the current of A-TEMPO increased in the low concentration range of VCM as compared to TEMPO. This method also was able to quantify VCM in the concentration range of 10–100 μM, which is an important concentration range for drug monitoring in blood.

## Abstract 2

An analytical method using nitroxyl radicals was able to quantify vancomycin (VCM) in the concentration range of 10–100 μM, which is an important concentration range for drug monitoring in blood.

## Graphical abstract

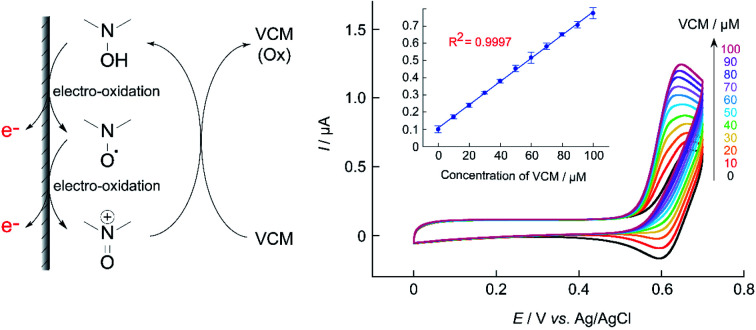

## Introduction

**Fig. 1:**
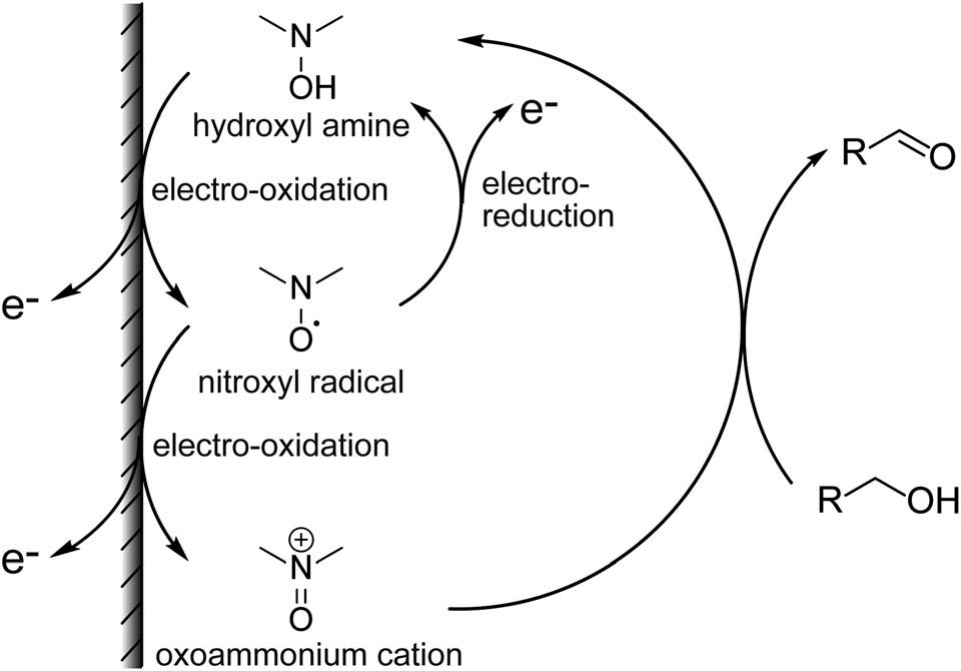
Electrochemical detection of alcohols using nitroxyl radicals.

Antibiotics are essential for the treatment of many infectious diseases, but they can cause side effects such as liver and kidney damage. In particular, the glycopeptide antibiotics vancomycin (VCM) and teicoplanin (TEIC) have been used widely because of their antimicrobial activity against most Gram-positive bacteria, including methicillin-resistant *Staphylococcus aureus* (MRSA).^1–3^ However, since VCM and TEIC can cause serious side effects, such as nephrotoxicity, VIII neuropathy, and redneck syndrome, therapeutic drug monitoring (TDM) in blood is important during treatment.^2–4^ Typical antibiotic analyses include immunological assays and separation methods (*e.g.*, HPLC, GC).^5,6^ Immunological methods, such as enzyme immunoassays (HEIA, EMIT), fluorescence immunoassays (FPIA),^7,8^ chemiluminescence immunoassays (CLIA),^9^ and latex agglutination turbidimetry (KIMS,^10^ PETINIA^11^) are simple and rapid assays. However, antibodies are affected differently by metabolites and concomitant drugs, resulting in different assay values.^8,12–14^ In contrast, liquid chromatography-tandem mass spectrometry (LC-MS/MS) often is used for the identification and quantification of molecules in various matrices due to its high specificity and accuracy.^7,15–17^ However, using LC-MS/MS for TDM has limitations such as a complicated procedure, low throughput, and expensive equipment. Due to these factors, the development of a simpler, less expensive, and more reliable quantification method is needed. Organic nitroxyl radicals, such as 2,2,6,6-tetramethylpiperidine 1-oxyl (TEMPO), are stable free radicals that can catalyze alcohol oxidation reactions when used with appropriate oxidants, and are therefore widely used in organic synthesis.^18–20^ In electrochemistry, hydroxy groups are oxidized by the application of an electric potential instead of an oxidant in basic aqueous solution and organic solvents.^21,22^ The increase in the anodic current depends on the concentration of alcohol in the solution, allowing the quantification of alcohols and other compounds with intramolecular hydroxy groups (Fig. 1).^23^ In general, enzymes are used as molecular recognition elements and signal conversion sites in electrochemical sensors. High specificity and time resolution can be obtained using enzymatic reactions. However, developing electrochemical sensors for drugs that are not involved in enzymatic reactions (*e.g.*, antibiotics) is basically not possible. Therefore, the use of organocatalysts, such as nitroxyl radical compounds, has been investigated for the determination of drugs. Electrochemical detection of compounds with intramolecular hydroxy groups is possible using highly active organocatalysts. The electrochemical detection of cyclosporin A in acetonitrile using organocatalysts has been reported.^24^

The present study involved the electrochemical detection of antibiotics with different structures using nortropine *N*-oxyl (NNO), which have a bicyclo skeleton and highly active, and TEMPO. The small molecule drugs, ethambutol, acyclovir, and meropenem, and the complex antibiotics, rifampicin, clarithromycin, azithromycin, streptomycin, TEIC, and VCM, were investigated for their compatibility with nitroxyl radicals. The electrochemical properties of the nitroxyl radicals (NNO and TEMPO) used for the experiments also were determined. Furthermore, 4-acetamido-2,2,6,6-tetramethylpiperidine 1-oxyl (A-TEMPO), which has a greater oxidation capacity than TEMPO due to its electron-withdrawing group, was used for the determination of VCM. This method was capable of quantifying VCM in the concentration range of 10–100 μM, which is an important concentration range for drug monitoring in blood.

## Experimental

### Materials and methods

**Fig. 2:**
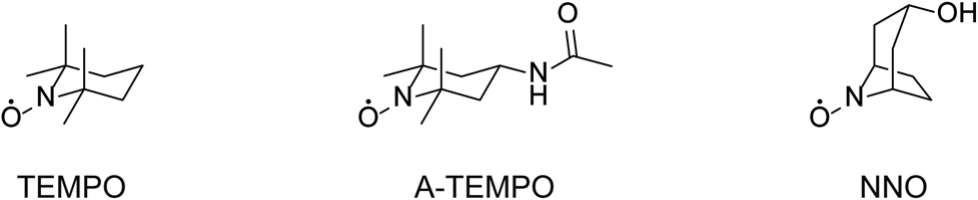
Chemical structures of TEMPO, A-TEMPO, and NNO.

The NNO was synthesized in one step from nortropine according to a previously published method.^25^ The following is a brief outline if the synthesis. Nortropine (5 g) and sodium tungstate(iv) dihydrate (1.25 g) were dissolved in purified water (50 mL), cooled to 4 °C, and stirred for 2 hours. Then, aqueous hydrogen peroxide (30%, 4.5 mL) was added, and the mixture stirred for another 6 hours. After that, the precipitate formed was collected by filtration, washed with purified water, and dried in a desiccator for 24 hours to obtain NNO as pale yellow crystals (yield: 36%). The TEMPO, A-TEMPO, acyclovir, rifampicin, clarithromycin, azithromycin, and streptomycin were purchased from Tokyo Kasei. VCM and meropenem were purchased from Wako Pure Chemicals. Ethambutol and TEIC were purchased from Cayman Chemical Company and Bio Vision, respectively. The reagents were used without purification. The nitroxyl radical derivatives used in this study are shown in Fig. 2.

### Apparatus

Cyclic voltammetry was performed using an electrochemical analyzer (ALS model 660B; BAS, Tokyo, Japan) in a conventional three-electrode cell consisting of a glassy-carbon electrode as the working electrode, a platinum wire as the counter-electrode, and an Ag/AgCl (3 mol L^−1^ KCl) reference electrode.

## Results and discussion

### Electrochemical determination of antibiotics

The TEMPO catalyzes oxidation of hydroxy groups upon application of an electric potential, and the resulting anodic current can be used to quantify alcohols and compounds with hydroxy groups.^25^ But TEMPO has low reactivity due to the large steric hindrance around the nitroxyl radical, its active site, resulting in insufficient anodic current in neutral aqueous solution.^26^ Therefore, NNO was developed as a catalyst that solves this problem by stabilizing the nitroxyl radical with a bicyclo structure. The NNO is more active than TEMPO due to the reduced bulkiness around the nitroxyl radical. Using this catalyst, non-enzymatic detection of glucose and lactate under physiological conditions and electro-oxidation of amines were possible, which were difficult to achieve with TEMPO.^25,27–29^

**Fig. 3:**
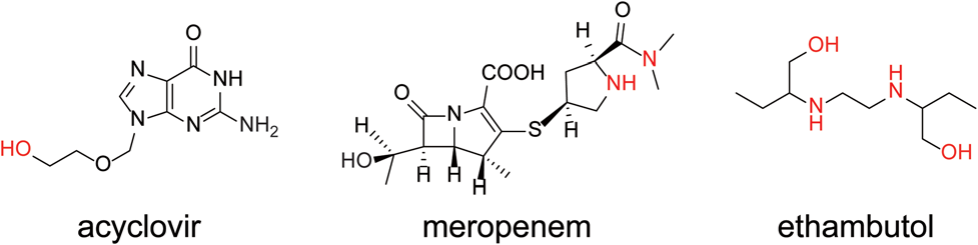
Chemical structures of acyclovir, meropenem, and ethambutol.

**Fig. 4:**
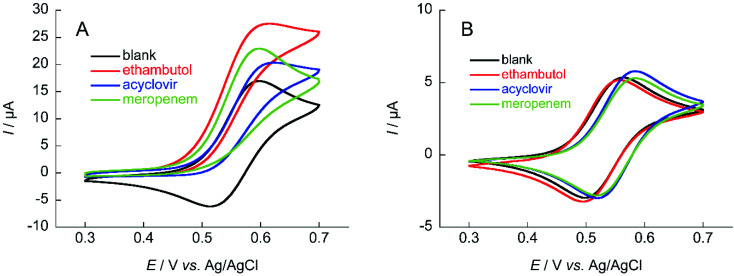
Cyclic voltammograms of (A) 1 mM NNO and (B) 1 mM TEMPO in the absence and presence of 1 mM ethambutol, acyclovir, and meropenem in 100 mM phosphate buffer (pH 7.4); scan rate: (A) 100 mV s^−1^, (B) 10 mV s^−1^.

First, the electrochemical responses to various antibiotics using NNO and TEMPO were compared. Low-molecular-weight compounds, such as ethambutol, acyclovir, and meropenem, were investigated (Fig. 3). The antibiotics (1 mM) and NNO or TEMPO (1 mM) were dissolved in 100 mM phosphate buffer (pH 7.4), and cyclic voltammetry measurements were performed (Fig. 4). Due to the high catalytic activity of NNO, the increase in current value due to the addition of substrate is largely observed at higher potential sweep rates (Fig. S1†). Since rapid measurement is preferable as the analysis method, cyclic voltammetry using NNO was performed with a scan rate of 100 mV s^−1^. The cyclic voltammogram of NNO without drug showed an anodic peak potential at +0.60 V *vs.* Ag/AgCl with a current value of 16.9 μA. The cathodic peak potential occurred at +0.52 V *vs.* Ag/AgCl. The cyclic voltammogram in the presence of a drug with a hydroxy group (*e.g.*, acyclovir) had an increase in the anodic peak current value while the cathodic current disappeared. This increase in the anodic peak current value was Δ*I*_p_ and used as an index of progress of electrocatalytic oxidation reaction.

The mechanism for the increase in Δ*I*_p_ is shown above (Fig. 1). For cyclic voltammetry, the nitroxyl radicals in the NNO are oxidized by the electrode to oxoammonium ions when the electrode potential sweeps to the high potential side. The oxoammonium ion has the ability to oxidize the hydroxy group of the drug molecule to an aldehyde group, and itself is reduced to a hydroxylamine. This hydroxylamine is again oxidized to oxoammonium by electrode oxidation and oxidizes a new drug molecule. As this cycle proceeds during one sweep of cyclic voltammetry, the resulting anodic current increases. Thus, if the reaction between the nitroxyl radical and the hydroxy group proceeds quickly, anodic current will be high. Similarly, when cyclic voltammetry sweeps from the high potential side to the low potential side, the cathodic current disappears (decreases) because the oxoammonium ion on the electrode surface is reduced by the hydroxy group.

The anodic peak current increases of NNO (Δ*I*_p_) in the presence of acyclovir, meropenem, and ethambutol during cyclic voltammetry were 2.7, 5.9, and 10.7 μA, respectively. The increase in Δ*I*_p_ was expected because of the hydroxy group in acyclovir. Reports have indicated that NNO electrolytically oxidizes not only hydroxy groups but also secondary and tertiary amines.^27^ Therefore, the cyclic voltammogram in the presence of meropenem also showed an increase in anodic current due to the progressive oxidation of amines. In addition, ethambutol showed a greater value for Δ*I*_p_ than did the other two drugs due to the presence of a primary alcohol and secondary amine. In contrast, TEMPO has a slow reaction rate with hydroxy groups in neutral aqueous solution, so an increase in Δ*I*_p_ could not be confirmed for these drugs at a scan rate of 100 mV s^−1^. However, it may be possible to observe the Δ*I*_p_ at a slower scan rate (Fig. S2†). Therefore, cyclic voltammetry using TEMPO was performed with a scan rate of 10 mV s^−1^. As a result, a slight increase in the current value was observed when acyclovir was applied.

**Fig. 5:**
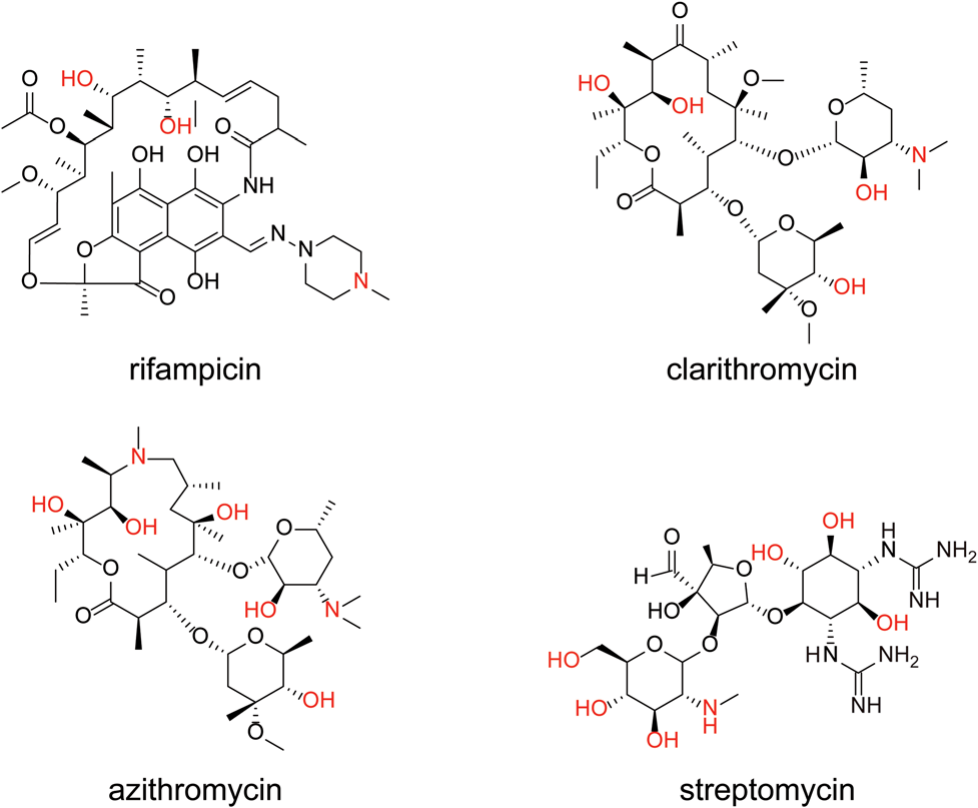
Chemical structures of rifampicin, clarithromycin, azithromycin, and streptomycin.

**Fig. 6:**
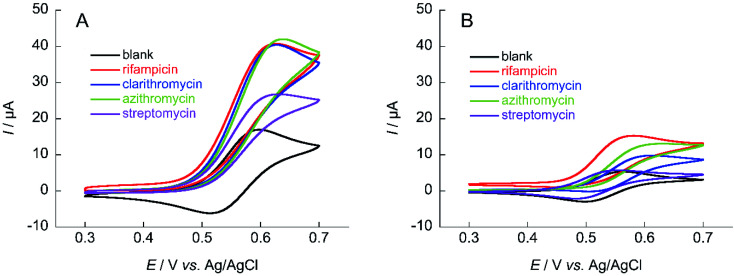
Cyclic voltammograms of (A) 1 mM NNO and (B) 1 mM TEMPO in the absence and presence of 1 mM rifampicin, clarithromycin, azithromycin, and streptomycin in 100 mM phosphate buffer (pH 7.4) containing 5% MeCN (rifampicin and azithromycin) or 30% MeCN (clarithromycin); scan rate: (A) 100 mV s^−1^, (B) 10 mV s^−1^.

The same measurements were performed on the medium-molecular-weight compounds, rifampicin, clarithromycin, azithromycin, and streptomycin. The structure of each antibiotic is shown in Fig. 5. Cyclic voltammograms of NNO and TEMPO in the presence of these drugs are shown in Fig. 6. These antibiotics have complex chemical structures with molecular weights ranging from 580 to 820, and they contain multiple hydroxy groups and secondary and tertiary amino groups. Therefore, the Δ*I*_p_ values were greater than those of the small-molecule drugs (see Fig. 4). Also, an increase in Δ*I*_p_ was observed when TEMPO was used, suggesting that some drugs could be analyzed electrochemically by lowering the scan rate (although it would take more time). (Note: for rifampicin, although an anodic current was observed even when rifampicin was used alone, the potential at that time is different than the potential range of this measurement, and the current value is small, so it was considered to have no effect.)

### Determination of vancomycin using TEMPO

**Fig. 7:**
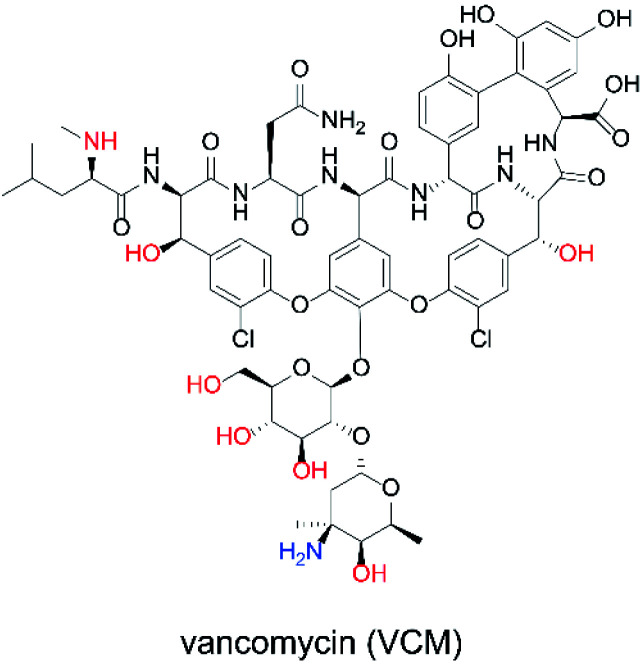
Chemical structure of VCM.

**Fig. 8:**
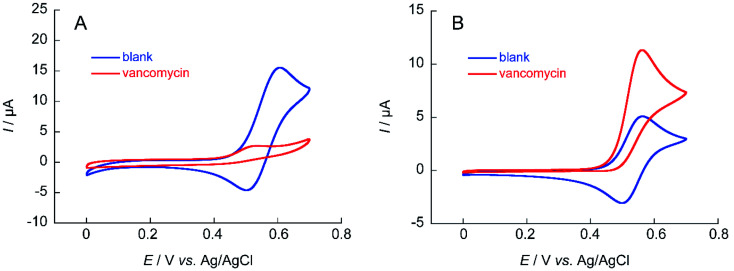
Cyclic voltammograms of (A) 1 mM NNO and (B) 1 mM TEMPO in the absence and presence of 1 mM VCM in 100 mM phosphate buffer (pH 7.4); scan rate: (A) 100 mV s^−1^, (B) 10 mV s^−1^.

The ability to obtain a high Δ*I*_p_ value in cyclic voltammetry measurements of NNO under physiological conditions in VCM was investigated. The chemical structure of VCM (Fig. 7) is more complex than those of the drugs shown in Fig. 5. Because it has six hydroxy groups and one secondary alcohol that can be electro-oxidized by nitroxyl radical catalysts, a significant increase in Δ*I*_p_ was expected. The cyclic voltammetry measurements were performed with a solution containing 1 mM of VCM and 1 mM of NNO (Fig. 8). In contrast, the anodic peak current of NNO found at +0.6 V was reduced by the presence of VCM. NNO was considered to be deactivated by irreversible binding of the primary amine in the VCM molecule to the oxoammonium ion produced by oxidation of the nitroxyl radical on the electrode.^30–32^ Nitroxyl radicals with a bicyclo skeleton are more active than TEMPO toward alcohols, but they are not applicable when the molecule to be analyzed has primary amines. When the same measurement was performed with TEMPO, an increase in Δ*I*_p_ was observed upon addition of VCM. Although TEMPO has low catalytic activity in neutral aqueous media, it is not deactivated by primary amines like the bicyclo-framework nitroxyl radical catalysts are, suggesting that the anodic current dependent on alcohol concentration can be obtained, even in the presence of primary amines.

Similar measurements were performed using teicoplanin (Fig. S3†). The structure of teicoplanin contains 10 hydroxy groups and one primary amine. Therefore, inactivation of NNO is expected as with VCM. Cyclic voltammetry of NNO in the presence of teicoplanin showed a slight decrease without an increase in Δ*I*_p_ and a disappearance of the cathodic current (Fig. S4†), due to inactivation by the primary amine in teicoplanin, as with VCM. In contrast, for TEMPO, an increase in Δ*I*_p_ was observed. These results support the inability to use the nitroxyl radical of the bicyclo skeleton for electrochemical detection where it is inactivated in the presence of primary amines. Thus, if a drug is electrochemically analyzed with a nitroxyl radical compound, a nitroxyl radical compound (NNO) with a bicyclo skeleton that has high catalytic activity should be used if no primary amine is present in the molecule to be analyzed. However, when primary amines are present, electrochemical analysis is possible by decreasing the scan rate using TEMPO, although the analysis takes longer and the current value decreases.

As described above, VCM contains primary amines, so although it is highly active, NNO cannot be used. Therefore, electro-oxidation reaction of VCM was investigated using nitroxyl radicals with a reduced scan rate using TEMPO and 4-acetamide TEMPO (A-TEMPO). The A-TEMPO, which like TEMPO has a piperidine skeleton, is not likely to be inactivated by amines in aqueous solution. In addition, for oxidation reactions by electrochemical methods, A-TEMPO has a greater oxidation capacity than TEMPO due to the electron-withdrawing effect derived from its acetamide group.^33^

**Fig. 9:**
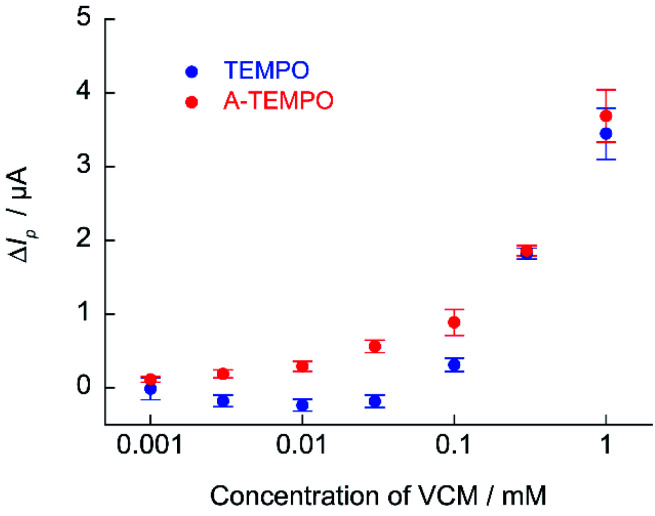
Calibration curve of Δ*I*_p_ against VCM concentration obtained from cyclic voltammetry of 1 mM A-TEMPO and TEMPO in 100 mM phosphate buffer (pH 7.4); scan rate: 10 mV s^−1^.

The calibration curve of Δ*I*_p_ against VCM concentration obtained from cyclic voltammetry of TEMPO and A-TEMPO in the presence of VCM is shown in Fig. 9. Results showed that Δ*I*_p_ increased with VCM concentration for both TEMPO and A-TEMPO catalysts in the pH 7.4 phosphate buffer solution. In particular, when A-TEMPO was used, detection was possible even in the concentration range of 10–100 μM, which is not possible with TEMPO.

A trough value of 10–20 μg mL^−1^ (6.73–13.46 μmol L^−1^) has been proposed as the therapeutic blood level during treatment with VCM.^34,35^ Maintaining a trough value of at least 10 μg mL^−1^ increase the efficacy of treatment of MRSA infection and avoids the risk of selecting low-susceptible strains.^36–38^ However, a trough value of 20 μg mL^−1^ or higher is associated with a high incidence of nephrotoxicity.^39–42^ For the safe use of VCM, maintaining a safe blood concentration range is important, and so monitoring is required to maintain drug efficacy and prevent problems. Since A-TEMPO can be used for electrochemical quantification of that range of VCM concentration, detailed investigations were conducted.

**Fig. 10:**
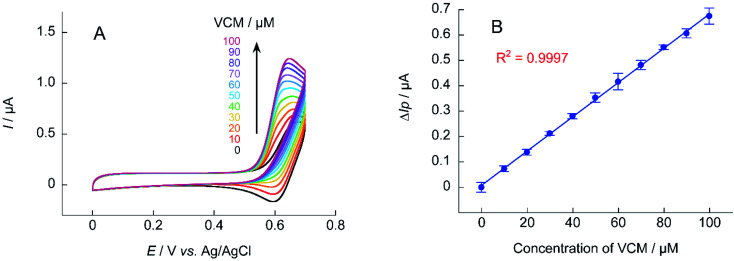
(A) Cyclic voltammograms of A-TEMPO (0.1 mM) in the absence and presence of VCM (10–100 mM) in 100 mM phosphate buffer (pH 7.4); scan rate: 10 mV s^−1^. (B) Calibration curve of Δ*I*_p_ for VCM concentration obtained from cyclic voltammetry.

For electrochemical measurements using nitroxyl radical catalysts, the increase in Δ*I*_p_ is smaller when the analyte is at a low concentration. The detection sensitivity decreases when the base anodic current derived from nitroxyl radical is relatively large (when the concentration of nitroxyl radical is high). Thus, if the sensitivity of the measurement device used is sufficient, decreasing the concentration of the nitroxyl radical catalyst will lower the base current and increase the response current signal. Quantification of VCM was attempted using 0.1 mM of A-TEMPO at a scan rate of 10 mV s^−1^ (Fig. 10). Results showed a concentration-dependent anodic current was obtained at 10–100 μM of VCM, producing a good calibration curve for VCM concentration.

### Comparison of the catalytic performance of NNO, TEMPO, and A-TEMPO

**Fig. 11:**
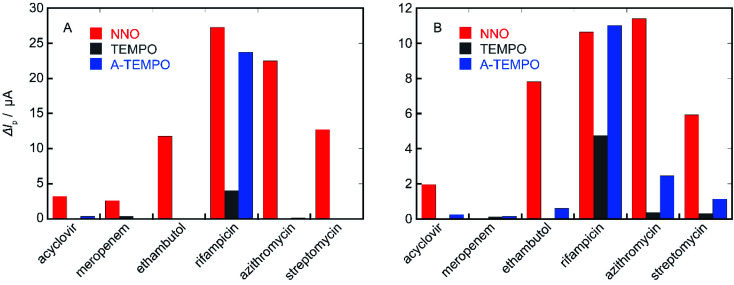
Δ*I*_p_ values of NNO, TEMPO, and A-TEMPO obtained using each antibiotic as a substrate; scan rate: (A) 100 mV s^−1^, (B) 10 mV s^−1^.

In order to confirm the results obtained from these investigation, the performance of the three types of catalysts NNO, TEMPO, and A-TEMPO was evaluated by Δ*I*_p_ using each antibiotic as a substrate (Fig. 11). As a result, it was clarified that A-TEMPO has higher catalytic activity than TEMPO, but NNO is also more active against antibiotics not containing primary amine. Therefore, it is important to select an appropriate catalyst according to the substance to be analyzed and use it under appropriate conditions.

## Conclusions

Electrochemical measurements were conducted using nitroxyl radical compounds against various antibiotics. Antibiotics with alcohols and secondary and tertiary amines were detectable with a high response current using NNO. In contrast, the analysis of antibiotics containing primary amines required a decrease in the scan rate and the use of TEMPO to obtain a response current. Results suggested that a wide range of antibiotics can be quantified by appropriately selecting the nitroxyl radical catalyst according to the molecular structure of the analyte. The use of A-TEMPO, which has a high electro-oxidation capacity, also allowed the quantification of VCM in the concentration range of 10–100 μM. This method should be applicable to a variety of other antibiotics, which should allow the development of a simple electrochemical measurement system.

## Author contributions

Conceptualization, K. Sa. and T. O.; methodology, K. Sa. and T. O.; software, K. Sa.; validation, K. Su., S. K. and M. K.; formal analysis, T. O. and Ka. S.; investigation, K. Su., S. K., M. K., T. D., Y. S. and Y. I.; resources, T. F., Y. K., K. Y. T. O. and K. Sa.; data curation, T. O. and K. Sa.; writing—original draft preparation, T. O. and K. Sa.; writing—review and editing, T. O. and K. Sa.; visualization, T. O. and K. Sa; supervision, K. Sa.; project administration, K. Sa.; funding acquisition, T. O. and K. Sa. All authors have read and agreed to the published version of the manuscript.

## Conflicts of interest

There are no conflicts to declare.

## Supplementary Material

RA-011-D1RA03681E-s001
