# Supplementary material for: Catalysis of electro-oxidation of antibiotics by nitroxyl radicals and the electrochemical sensing of vancomycin
Source: RSC Adv. 2021 Jun 18;11(35):21622–8. doi: 10.1039/d1ra03681e (PMC9034136; doi:10.1039/d1ra03681e)
Supplement: RA-011-D1RA03681E-s001 [file RA-011-D1RA03681E-s001.pdf]

Electronic Supplementary Material (ESI) for RSC Advances.
This journal is © The Royal Society of Chemistry 2021

Supplementary Information

Catalysis of electro-oxidation of antibiotics by nitroxyl radicals and the electrochemical sensing of vancomycin

Tetsuya Ono,*[a] Kyoko Sugiyama,[b] Sachiko Komatsu,[b] Masayuki Kumano,[c] Kentaro Yoshida,[a] Takenori Dairaku,[a] Tsutomu Fujimura,[b] Yusuke Sasano,[c] Yoshiharu Iwabuchi,[c] Yoshitomo Kashiwagi [a] and Katsuhiko Sato *[b,d]

1) School of Pharmaceutical Sciences, Ohu University, 31-1 Misumido, Tomita-machi, Koriyama, Fukushima 963-8611, Japan; t-ono@pha.ohu-u.ac.jp (T.O.); k-yoshida@pha.ohu-u.ac.jp (K.Y.); y-kashiwagi@pha.ohu-u.ac.jp (Y.K.)
2) Faculty of Pharmaceutical Science, Tohoku Medical and Pharmaceutical University, 4-4-1 Komatsushima, Aoba, Sendai, Miyagi 981-8558, Japan; kyoko.sugiyama@tohoku-mpu.ac.jp (K.Su.); sachicom@tohoku-mpu.ac.jp (S.K.); tfujitsu@tohoku-mpu.ac.jp (T.F.)
3) Graduate School of Pharmaceutical Sciences, Tohoku University, 6-3 Aoba, Aramaki, Aoba-ku, Sendai 980-8578, Japan; masayuki.kumano.p2@dc.tohoku.ac.jp (M.K.), ysasano@tohoku.ac.jp (Y.S.), iwabuchi@mail.pharm.tohoku.ac.jp (Y.I.)
4) Department of Creative Engineering, National Institute of Technology, Tsuruoka College, 104 Sawada, Inooka, Tsuruoka, Yamagata 997-8511, Japan; satok@tohoku-mpu.ac.jp (K.Sa.)

Correspondence:

Tetsuya Ono (t-ono@pha.ohu-u.ac.jp); Tel.: +81-24-932-8931

Katsuhiko Sato (satok@tohoku-mpu.ac.jp); Tel.: +81-22-727-0079

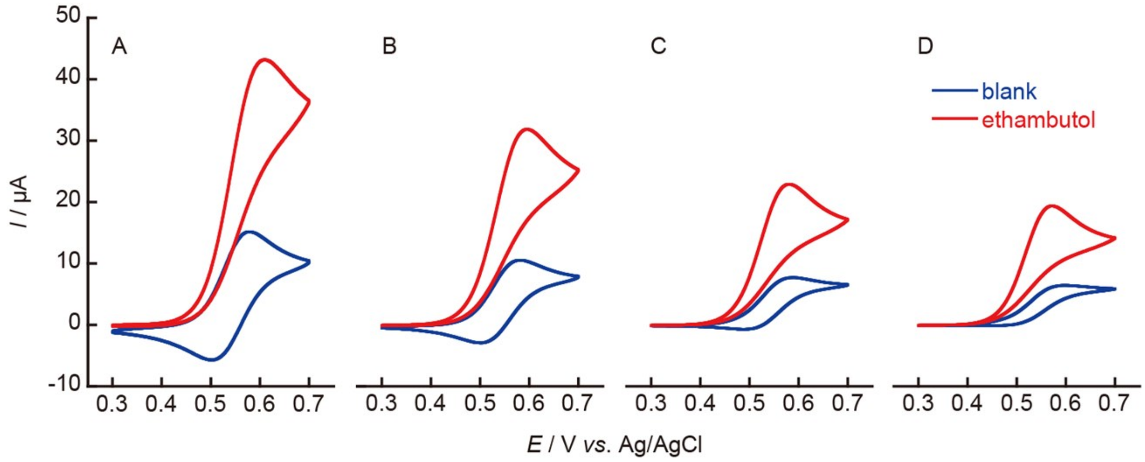

Fig. S1 Cyclic voltammograms of 1 mM NNO in the presence and absence of 10 mM ethambutol in 100 mM phosphate buffer (pH 7.4); scan rate (A) 100 mV s^{-1}, (B) 50 mV s^{-1}, (C) 20 mV s^{-1}, (D) 10 mV s^{-1}.

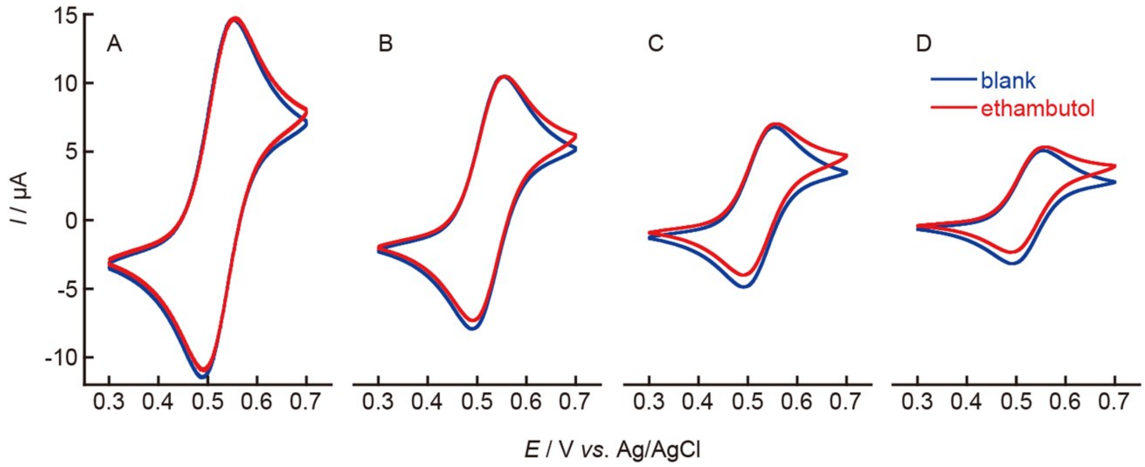

Fig. S2 Cyclic voltammograms of 1 mM TEMPO in the presence and absence of 10 mM ethambutol in 100 mM phosphate buffer (pH 7.4); scan rate (A) 100 mV s^{-1}, (B) 50 mV s^{-1}, (C) 20 mV s^{-1}, (D) 10 mV s^{-1}.

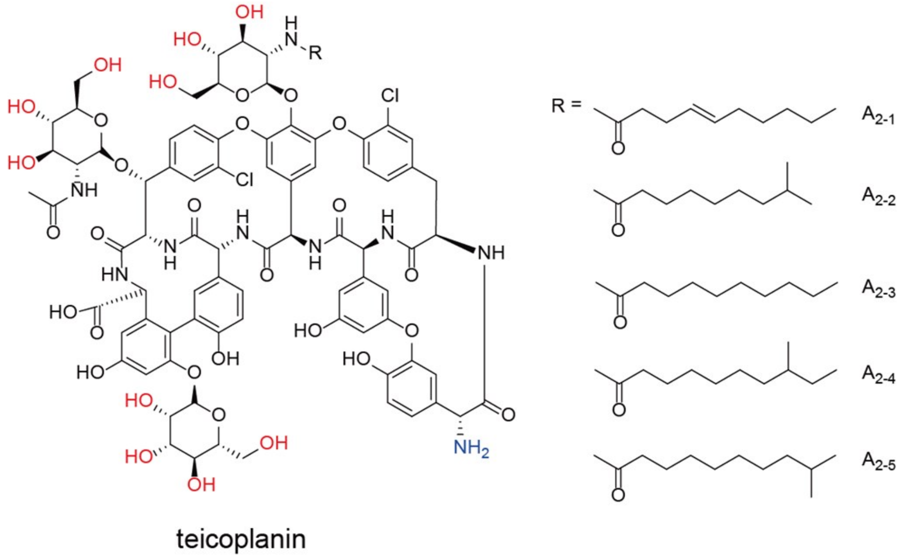

Fig. S3 Chemical structures of teicoplanin.

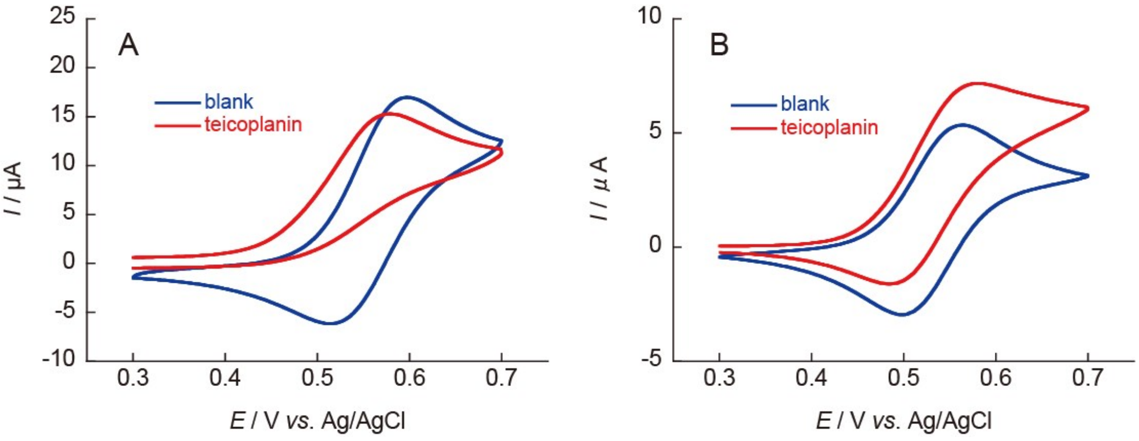

Fig. S4 Cyclic voltammograms of (A) 1 mM NNO and (B) 1 mM TEMPO in the presence and absence of 1 mM teicoplanin in 100 mM phosphate buffer (pH 7.4); scan rate (A) 100 mV s^{-1}, (B) 10 mV s^{-1}.
